# Supplementary material for: Depression and cancer outcomes in resource-limited settings: A cross-sectional analysis of treatment uptake and survival among metastatic breast cancer patients in Lagos, Nigeria
Source: PLOS Glob Public Health. 2026 Mar 31;6(3):e0005969. doi: 10.1371/journal.pgph.0005969 (PMC13037999; doi:10.1371/journal.pgph.0005969)
Supplement: S1 Table — (DOCX) [file pgph.0005969.s001.docx]

**S1 Table.** Kaplan-Meier Survival Probabilities by Depression Status

| **Time Point** | **Survival % (95% CI)** | | **Difference** |
| --- | --- | --- | --- |
|  | **CSD Group** | **Non-CSD Group** |  |
| 200 days | 89.8% (77.8-95.9) | 91.1% (86.4-94.4) | -1.3% |
| 400 days | 87.8% (75.2-94.4) | 88.9% (83.8-92.5) | -1.1% |
| 600 days | 85.7% (72.2-93.0) | 86.2% (80.7-90.3) | -0.5% |
| 800 days | 85.7% (72.2-93.0) | 85.3% (79.5-89.6) | +0.4% |
