# Supplementary material for: Depression and cancer outcomes in resource-limited settings: A cross-sectional analysis of treatment uptake and survival among metastatic breast cancer patients in Lagos, Nigeria
Source: PLOS Glob Public Health. 2026 Mar 31;6(3):e0005969. doi: 10.1371/journal.pgph.0005969 (PMC13037999; doi:10.1371/journal.pgph.0005969)
Supplement: Attachment — Submitted filename: Response to Reviewer 2.docx [file pgph.0005969.s003.docx]

**POINT-BY-POINT RESPONSE TO REVIEWERS’ COMMENTS**

Manuscript ID: PGPH-D-26-00350

*Depression and Cancer Outcomes in Resource-Limited Settings: A Cross-Sectional Analysis of Treatment Uptake and Survival Among Metastatic Breast Cancer Patients in Lagos, Nigeria*

Dear Editor-in-Chief and Editorial Board,

We sincerely thank the Editor and both Reviewers for their constructive and insightful evaluations of our manuscript. The comments have been invaluable in strengthening the scientific rigor, interpretive depth, and contextual grounding of this work. We have carefully and comprehensively addressed every point raised. All revisions in the revised manuscript are indicated in red text for ease of identification. Below, we present our detailed point-by-point responses. Reviewer comments are presented in black, and our responses follow in red regular text.

Respectfully,

Oluwaseun Adebayo Bamodu, MD, MS, PhD

*On behalf of all co-authors*

# **POINT-BY-POINT RESPONSE TO REVIEWER #2**

*(Reviewer: Dr. Taiwo Olufemi Abiona)*

**Comment 1:** In Table 2, the p-values for BDI-II categories are redundant with the overall group difference; consider simplifying this.

**Response 1:** We appreciate this observation. The reviewer is correct that the individual BDI-II category p-values are inherently redundant with the overall CSD/non-CSD group comparison, since the categories are deterministically defined by the BDI-II ≥20 threshold. Table 2 has been revised to retain only the overall BDI-II score comparison (p<0.001) and remove the redundant p-value for the categorical distribution. A footnote has been added clarifying: “BDI-II severity categories are deterministically defined by the CSD threshold (≥20); the p-value represents the continuous score comparison.”

Please see revised Table 2 in our revised manuscript.

**Comment 2:** The hazard ratio (0.086) of lung metastasis is striking. Consider presenting as “91% lower hazard of the event compared with the reference group” for clarity.

**Response 2:** We thank the reviewer for this excellent suggestion for improved interpretability. We have adopted this recommendation and now present the finding as: “Lung metastasis was associated with a 91.4% lower hazard of mortality (adjusted HR 0.086, 95% CI 0.019–0.397, p=0.002)” in both the Results text and an interpretive footnote to Table 4. We have also expanded the discussion of this counterintuitive finding with multiple explanatory hypotheses (ascertainment bias, differential access, immortal time bias) as detailed in our response to Reviewer 1, Comment 5(d).

Please see the revised Results (lines 314–322) and Discussion sections of the revised manuscript.

**Comment 3:** For the mental health stigma and underreporting, the authors acknowledged stigma, but the implications of this have not been fully explored. If patients underreport depressive symptoms, the CSD group may be misclassified, where some truly depressed patients are categorised as being in the non-CSD group, biasing the results. I suggest adding this to the manuscript’s limitations section.

**Response 3:** We are grateful for this incisive observation, which identifies a critically important source of potential bias. This point has been substantively incorporated into the Limitations section as follows:

We now state: “A particularly important limitation relates to potential misclassification bias arising from mental health stigma and underreporting. In the Nigerian sociocultural context, where mental health stigma remains substantial (Cuijpers, 2023), patients may underreport depressive symptoms on self-administered instruments. This would result in differential misclassification, whereby truly depressed patients scoring below the BDI-II ≥20 threshold would be classified into the non-CSD group, effectively diluting the CSD group and attenuating any true association between depression and outcomes toward the null. This non-differential misclassification of the exposure represents a bias toward the null hypothesis and may partly explain the absence of observed associations. The potential magnitude of this bias is difficult to quantify without a gold-standard clinical interview comparison, but the documented prevalence of mental health stigma in Nigerian healthcare settings suggests it may be non-trivial.”

This addition strengthens the limitations section by explicitly connecting the acknowledged stigma to its specific epidemiologic consequence (misclassification bias toward the null) and the direction of its expected effect on our findings.

Please see the revised Limitations section (lines 429–449 of the revised manuscript).
